# Potential mechanisms of exercise for relieving inflammatory pain: a literature review of animal studies

**DOI:** 10.3389/fnagi.2024.1359455

**Published:** 2024-02-08

**Authors:** Minmin Wu, Wenjing Song, Mei Zhang, Lili Teng, Qiang Tang, Luwen Zhu

**Affiliations:** ^1^Department of Rehabilitation Medicine, Heilongjiang University of Chinese Medicine, Harbin, China; ^2^The Second Affiliated Hospital of Heilongjiang University of Chinese Medicine, Harbin, China

**Keywords:** inflammatory pain, exercise, animal model, chronic pain, biological mechanisms

## Abstract

Inflammatory pain (IP) is one of the most prevalent and intractable human conditions, and it leads to progressive dysfunction and reduced quality of life. Additionally, IP is incredibly challenging to treat successfully with drugs or surgery. The development of IP is complex and multifactorial, and peripheral and central sensitization may influence chronicity and treatment resistance in IP. Understanding the mechanisms underlying IP is vital for developing novel therapies. Strong evidence suggests that exercise can be a first-line relief for patients with IP during rehabilitation. However, the mechanisms through which exercise improves IP remain unclear. Here, we reviewed the current animal experimental evidence for an exercise intervention in IP and proposed biological mechanisms for the effects of synaptic plasticity in the anterior cingulate cortex, endocannabinoids, spinal dorsal horn excitability balance, immune cell polarization balance, cytokines, and glial cells. This information will contribute to basic science and strengthen the scientific basis for exercise therapy prescriptions for IP in clinical practice.

## 1 Introduction

Pain is an unpleasant sensory and emotional experience that performs an important physiological function in the body by protecting body tissues from harmful stimuli (Zhang X. et al., 2023; Zhang Z.-Y. et al., 2023). However, prolonged severe pain appears to be a major consideration for return to work and quality of life for at least 5 years after discharge (Wang et al., 2023). Inflammation is a different biochemical reaction of the immunological, sensory, and neural systems to infection, tissue injury, and irritation (Varrassi et al., 2019). Peripheral tissue damage and inflammation are the main causes of inflammatory pain (IP), a common form of chronic clinical pain. Alterations in immune cell buildup and recruitment and the secretion of inflammatory factors activate nociceptors leading to pain (Hwang et al., 2019). Although pain due to acute inflammation can protect against harmful stimuli and promote the healing of damaged tissues, chronic pain is maladaptive and lasts beyond the normal healing process (Vergne-Salle and Bertin, 2021).

IP is a major health problem globally, affecting 350 million people worldwide, and its prevalence is increasing (Malange et al., 2022). This increasing prevalence is associated with a lack of effective treatments. Pain sensitization is thought to be a critical process in IP, in which chemical mediators responsible for tissue inflammation act on injured nerve terminals to reduce neuronal excitation thresholds and sensitize afferent discharge rates, resulting in abnormal pain and hyperalgesia (Ohashi et al., 2023). Typically, patients are treated with MAPK, chemokine, and cytokine inhibitors (Huang et al., 2021). However, these drugs can cause adverse consequences, like infection, after prolonged treatment and hinder inflammation healing. Nonpharmacological therapies, such as physical exercise, are cost-effective approaches that are easy to apply and have few adverse effects (Sartori et al., 2020). In recent years, the European Alliance of Associations for Rheumatology (EULAR) has recommended the use of physical exercise for IP-related conditions (Rausch Osthoff et al., 2018), such as inflammatory arthritis and inflammatory musculoskeletal disorders.

Although exercise intervention has rapidly evolved as a clinical strategy for addressing various conditions, its primary use in reducing IP is relatively young. Most treatments only reduce inflammation, while pain persists, and existing studies involving mechanisms and pathways are incompletely described, requiring the use of a variety of animal chronic pain models and techniques to better understand them. This review describes the current state of animal models examining exercise interventions for IP, analyzes the potential mechanisms by which exercise has anti-inflammatory and analgesic effects, and suggests that exercise may be a clinical treatment for IP-related diseases.

## 2 Nociceptor and the immune system in IP

**Figure 1:**
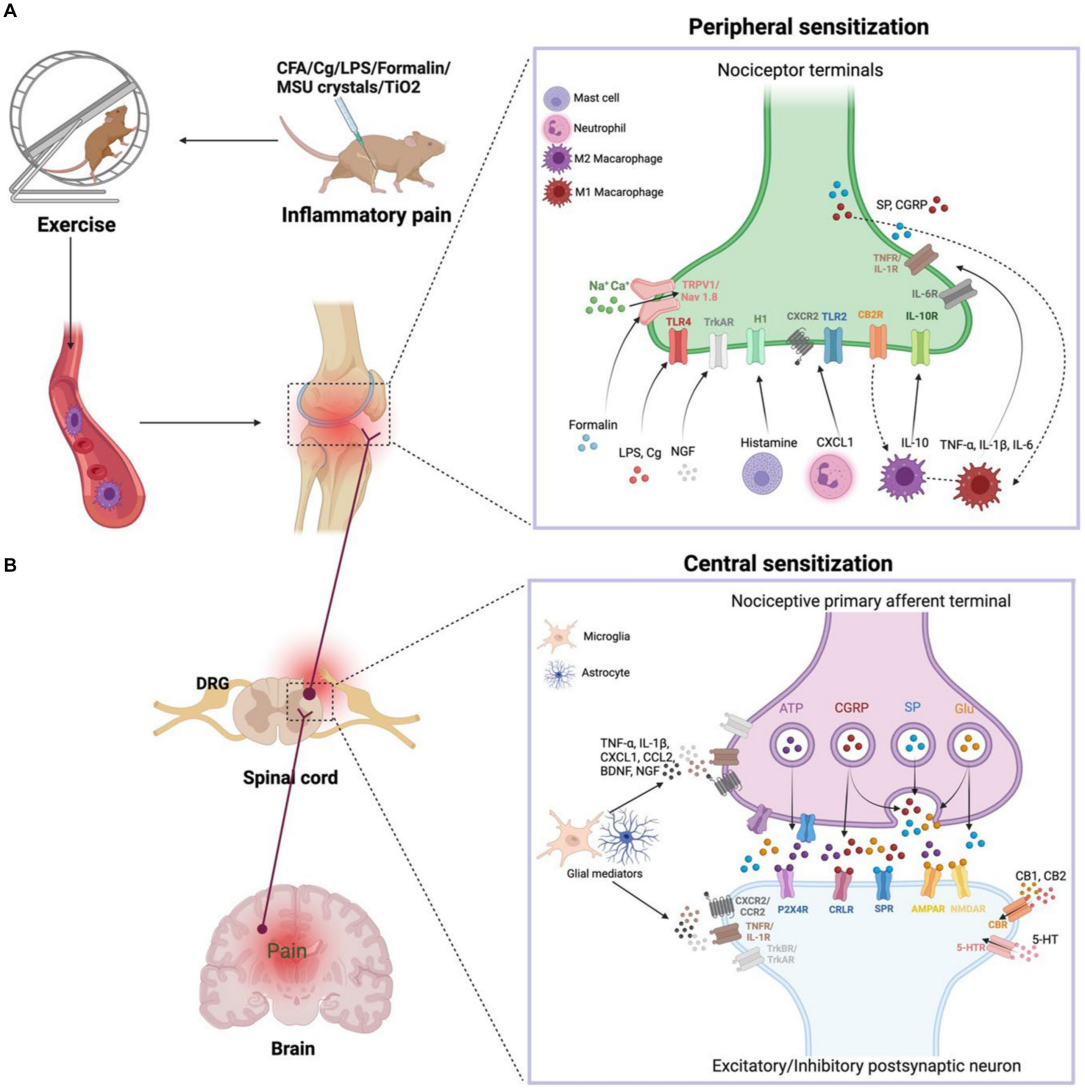
**(A)** Peripheral sensitization. During inflammation, tissue-resident and recruited immune cells secrete molecular mediators that act on the peripheral nerve terminals of nociceptor neurons, producing pain sensitization. Activation of nociceptor also releases SP and CGRP involved in neurogenic inflammation production. Exercise decreases the nociceptive hypersensitivity response and modulates pro-inflammatory cytokine levels. In addition, exercise achieves anti-inflammatory pain relief by modulating the balance of M1 and M2 macrophages in the immune system. **(B)** Central sensitization. During nerve injury or chronic pain, nociceptor neurons express inflammatory mediators and release them into the spinal cord, including ATP, CGRP, SP, and Glu. Activated microglia produce TNF-α, IL-1β, CXCL1, CCL2, BDNF, NGF. These inflammatory mediators modulate excitatory and inhibitory synaptic transmission, leading to central sensitization and chronic pain state is enhanced. However, exercise downregulates pro-inflammatory factors and produces anti-inflammatory factors such as IL-10, 5-HT, CB, opioids, which normalize neuroinflammation, synaptic plasticity and abnormal chronic pain.

IP is regulated by the bidirectional communication between the immune system and nociceptor neurons (Silva et al., 2017). Primary hyperalgesia results from the sensitization of nociceptor nerve terminals, while secondary hyperalgesia is caused by plasticity changes in the CNS. We will briefly describe the progression of IP from the periphery to the center, which contributes to the understanding of exercise relief of IP (Figure 1).

### 2.1 Peripheral sensitization

When tissue injury occurs, immune cells infiltrate into the injured tissue, which causes inflammation. Immune cells secrete various inflammatory substances, including CXCL1, CCL2, TNF-α, IL-6, NGF, IL-1β, and histamines, that act on ionotropic receptors, GPCRs, and TKRs on peripheral nociceptor nerve ends. These receptors mediate a signaling cascade that leads to peripheral sensitization and pain. The production of Ca^2+^ and cAMP activates the p38-MAPK and JNK signaling pathways, which regulate transient receptor potential ion channels (TRPV1 and TRPV4) and sodium channels (Nav1.7 and Nav1.8) (Matsuda et al., 2019). The activation of ion channels increases neuronal firing, leading to sustained peripheral sensitization. Additionally, the accumulation of some immune cells, such as mast cells, in chronic inflammatory conditions leads to chronic pain, and inflammatory factors produced by macrophages and monocytes act on nociceptor neurons to amplify pain. Gong et al. (2016) have suggested that exercise lowers pain in a rat of neonatal incisional surgery by inhibiting p38-MAPK activation and IL-1β and TNF-α secretion.

### 2.2 Central sensitization

The peripheral afferent nerves are used to carry IP signals. When nerve damage or persistent pain, nociceptor neurons release inflammatory substances (glutamate, adenosine triphosphate, SP, and CGRP) and release them into the spinal cord via central nerve terminals (Ji et al., 2018). Activation of the NMDAR by glutamate enhances synaptic efficacy and Ca^2+^ input, stimulating intracellular signaling networks that begin and prolong central sensitization. However, regular exercise inhibits the development of chronic pain through lowering the CNS’s phosphorylation of the NMDAR NR1 component (Sluka et al., 2013). Collagenous arthritis pain is linked to a mechanical hypersensitivity response in which innervated neurons enhance CGRP expression, activate ERK signaling, and lead to more pronounced microglial proliferation in the dorsal horn (Nieto et al., 2015).

Glial cells are central nervous cells that can be activated in a manner similar to that of peripheral macrophages (Pinho-Ribeiro et al., 2017). Glial cell activation is a vital factor in IP development. Activation of glial cells in IP is characterized by changes in neuroglial shape and elevation of the expression of markers such as IBA1 in microglia and GFAP in astrocytes. Neuroglia in the spinal cord express pro-inflammatory substances (TNF-α and IL-1β), neurotrophic factors (BDNF and NGF), and chemokines (CXCL1 and CCL2), which contribute to IP. CX3CL1 receptor is specifically produced in microglia in a p38-MAPK-dependent way and activates TNF-α (Maha et al., 2009). TNF-α activates astrocyte signaling in an ERK- and JNK-dependent manner that ultimately results in chronic pain (Gao and Ji, 2010). Immobilization causes an increase in inflammatory factors (SP and CGRP) and enhances nociceptive sensitization in rats after a fracture (Guo et al., 2014). Conversely, voluntary exercise reduces inflammatory nociceptive hyperalgesia induced by hindlimb immobilization in rats, inhibits the central sensitization of the spinal cord and the alteration of primary neurons caused through immobility, and reduces the number of CGRP-positive cells in the DRG and the spinal cord’s secretion of CGRP (Ishikawa et al., 2021). Moreover, physical exercise promotes the secretion of IL-10 by glial cells (Ferretti et al., 2005). We hypothesize that physical exercise normalizes neuroimmune signaling in the CNS, thereby preventing and reversing the development of hyperalgesia.

## 3 Mechanisms by which exercise reduces IP

Examining IP experimental models and different techniques for assessing pain has improved our understanding of peripheral and central pain mechanisms. Here, we summarize the animal models of exercise as a treatment for IP. These include IP models produced by complete Freund’s adjuvant (CFA), lipopolysaccharide, carrageenan (Cg), and formalin; a titanium dioxide (TiO_2_)-induced chronic arthritis model; a monosodium urate crystal-induced acute gout model; and a Secreted Protein Acidic Rich in Cysteine (SPARC)-null low back pain mice. More information on research examining exercise relief for IP is listed in Table 1.

**Table 1:** Regulation of the anti-inflammatory and analgesic effects of exercise.

Authors	Animal model	Exercise method	Exercise parameter	Pain measurement	Involved in pathways	Test site	Effect of exercise
Zhou et al. (2022)	CFA mice	Aerobic exercise (semivoluntary)	30 min/day, 15 days	Mechanical allodynia, hyperalgesia behavior	—	ACC	Serotonin ↑ Administration of 5-HT1A and 5-HT7 receptor antagonist affects exercise effect
Ludtke et al. (2020)	CFA mice	High-intensity swimming exercise	30 min/day, 7 days; 37°C	Mechanical hyperalgesia	—	Central (spinal cord), peripheral (hind paw)	CB1 (AM281), CB2 (AM630), AEA, 2-AG ↑
Pitcher et al. (2017)	CFA rat	Voluntary exercise	2 h/day, 12 days	Thermal hypersensitivity	—	Hind paw	—
de Azambuja et al. (2021)	Cg-induced acute muscle hyperalgesia rat	Swimming	50 min/day, 15 days; 31°C	Mechanical muscle hyperalgesia	PPARγ	Right gastrocnemius muscle	IL-1β, F4/80^+^ − CD11c^+^ ↓ IL-10, F4/80^+^ − CD206^+^ ↑
de Azambuja et al. (2020)	Cg-induced acute muscle pain rat	Swimming	40 min/day, 10 weeks; 31°C	Mechanical muscle hyperalgesia	PPARγ	Right gastrocnemius muscle	CINC-1 ↓
Dos Santos et al. (2019)	Cg-induced inflammatory muscle pain mice	Swimming	3 weeks, 15 min/day (1 week), 30 min/day (2 weeks), 45 min/day (3 weeks); 37°C	Tactile allodynia, thermal hyperalgesia	AEA-CB2 receptors	Right gastrocnemius muscle, spinal cord	Spinal cord: AEA, CB2 ↑ IBA1 (microglial marker), TNF-α ↓ Muscle: TNF-α, IL-1β, ↓
Nakabayashi et al. (2016)	A mixture of kaolin and Cg-induced arthritis	Continuous passive motion	60/day, 56 days	Mechanical hyperalgesia	—	Spinal cords (L2-3, L4-5)	CGRP ↓
King-Himmelreich et al. (2017)	Formalin-induced inflammatory nociception mice	Aerobic exercise	1 h/day	—	MAPK-*c*-Fos, AMPK	Spinal cord, serum	CB1, AEA ↑
Kuphal et al. (2007)	Formalin-induced rat	Swimming exercise	90 min/day, 9 days; 37°C (water temperature)	Thermal hyperalgesia, cold allodynia	—	Paw	Licking and flinching magnitude ↓
Jablonski et al. (2020)	MSU crystals-induced acute gout mice	Physical activity (running)	45 min/day, 14 days; 11 m/min, 15 m/min	—	NF-κB	Serum, joint	IL-1β, CXCL1, TLR2 ↓ F4/80^+^, MPO^+^↓
Feldman-Goriachnik et al. (2022)	LPS-induced mice	Free wheel running	7 days	Mechanical hyperalgesia	—	Hind paw, DRG	GFAP, SGCs, neuron ↓
Lee et al. (2022)	SPARC-null LBP female/male mice	Voluntary running	6 months	Mechanical sensitivity, cold sensitivity	—	Hind paw	Female: CXCL1 (IL-8 receptor), CXCL5 ↓ IL-1Ra, CXCR1, CXCR2, CD206 ↑ Male: M-CSF, VEGF, CXCR1, ITGAM ↑
Guirro et al. (2022)	TiO_2_-induced chronic arthritis female mice	Voluntary running	8 weeks	Mechanical hyperalgesia	—	Joint, EDL muscle	MDA, TNF-α, IL-6↓ Synovial hyperplasia, leukocyte infiltration and vascular proliferation ↓
Takahara-Yamauchi et al. (2021)	Formalin-induced rat	Voluntary exercise	11 days	Mechanical allodynia and hyperalgesia	BDNF/TrkB	Hind paw, spinal cord (right dorsal horn)	IBA1, KCC2 ↓ TrkB ↑
Sakamoto et al. (2023)	MIA-induced knee OA rat	Regular walking exercise	60 min/day, 5 days/week, 6 weeks	Pressure pain threshold	—	Right knee joint	IL-4, IL-10, CD206- ↑ CD68-, CD11c-, IL-1β ↓
Deng et al. (2023)	DMM surgery induces OA in rats	Moderate treadmill exercise	15 m/min, 30 min/day, 5 days/week, 6 weeks	Mechanical and heat pain	PI3K/AKT/mTOR	Paw	Col2a1 ↑ TNF-α, IL-6 MFN2, MMP13 ↓

CFA, complete Freund’s adjuvant; ACC, anterior cingulate cortex; CB, cannabinoid; SPARC, Secreted Protein Acidic Rich in Cysteine; LBP, low back pain; EDL, extensor digitorum longus; Cg, carrageenan; MSU, monosodium urate; MIA, monosodium iodoacetate; OA, osteoarthritis; DMM, destabilization of the medial meniscus; MFN2, mitofusin 2; MMP13, matrix metallopeptidase 13; AEA, anandamide; 2-AG, 2-arachidonyl glycerol; PPARγ, peroxisome proliferator-activated receptor gamma; CINC-1, cytokine-induced neutrophil chemoattractant 1; LPS, lipopolysaccharide; GFAP, glial fibrillary acidic protein; DRG, dorsal root ganglia; SGCs, satellite glial cells; 5-HT, 5-hydroxytryptamine; IL, interleukin; TNF-α, tumor necrosis factor alpha; CGRP, calcitonin gene-related peptide; MAPK, mitogen-activate protein kinase; NF-κB, nuclear transcriptional factor-κB; CXCL, chemokine C-X-C motif chemokine ligand; TLR2, toll-like receptor 2; VEGF, vascular endothelial growth factor; MDA, malondialdehyde.

Understanding the biological mechanisms underlying the link between inflammatory mediators and pain perception will provide an essential basis for prescribing exercise interventions for IP (Figure 1). Exercise modulates pro-inflammatory cytokine levels, decreases nociceptive hypersensitivity, attenuates behavioral pain responses, and exerts specific anti-inflammatory and analgesic properties. Mechanisms of the effect of exercise relief in IP include the control of synaptic plasticity in the anterior cingulate cortex (ACC), regulation of endocannabinoids (eCBs), alteration of excitability balance in the spinal dorsal horn (SDH), regulation of immune cell polarization balance, alteration of cytokines, and role of glial cells.

### 3.1 Synaptic plasticity in the ACC

The ACC is a key region of the limbic system, and human and animal studies have consistently shown that excitation or enhancement of the ACC plays an essential role in the emotional anxiety, behavioral sensitization, and pain unpleasantness associated with chronic pain (Xiao et al., 2021). Both acute and persistent pain cause the ACC to become active (Bliss et al., 2016). ACC neurons receive sensory projections from the thalamus and somatosensory cortex, and the ACC modulates pain and pain empathy through its projections to other subcortical areas and the spinal cord. The sensory component of pain is reduced after surgical ablation of the ACC (cingulotomy and cingulotomy) or blockade of the main projection pathway of the ACC (Xiao et al., 2021). Additionally, both human brain imaging and electrophysiological measurements of ACC neurons in animals have demonstrated that nociceptive stimuli activate neurons in the ACC, and animal models of pain indicate that inhibition of central plasticity in the ACC produces an analgesic effect (Koga et al., 2015).

Long-term potentiation (LTP) and long-term depression (LTD) are forms of synaptic plasticity that have been extensively studied in the areas of learning and memory. Evidence has been presented that functional abnormalities of LTP and LTD in the dorsal horn and cortical areas of the spinal cord (ACC) are causally related to chronic pain (Bliss et al., 2016). Aerobic exercise modulates changes in LTD and LTP in synaptic plasticity (Zhou et al., 2022). The coexistence of two kinds of LTP (pre-LTP- and post-LTP) in the ACC provides a possible mechanism for the development of chronic pain. The stimulation of GluK1-containing kainate receptors, protein kinase A, HCN, and adenylyl cyclase type 1channels, are essential for pre-LTP. By contrast, post-LTP needs the stimulation of postsynaptic NMDAR and AMPAR (Koga et al., 2015). Exercise inhibits HCN and NMDAR plasticity, which results in analgesic effects. Moreover, NMDAR-dependent signaling in neurons can be independently maintained in a CFA-induced IP mouse model, identifying a feed-forward signaling cascade in which NMDAR activation promotes pPKCγ production in neurons and pERK2 and IL-1β induction in activated astrocytes (Weyerbacher et al., 2010). Zhou et al. (2022) indicated that the ACC region is crucial for the ability of regular exercise to relieve IP. Exercise’s positive effects on pain-related behavior partially restore LTP occlusion through controlling synaptic plasticity in the ACC and promoting serotonin release. Moreover, pharmacological and genetic-based behavioral investigations have discovered that signaling through the 5-HT7 and 5-HT1A receptors in the ACC is significantly ameliorated when exercise modulates IP (Zhou et al., 2022).

Although exercise is used as a non-invasive treatment for chronic pain by modulating the ACC, the molecular and cellular mechanisms behind it remain unclear. As we utilize these treatments to manage IP, we must understand which neurotransmitters and signaling pathways are critical in the ACC and other regions including the insular cortex, primary somatosensory cortex, secondary somatosensory cortex, and prefrontal cortex. Further research is necessary to develop more specific factors to study the mechanisms, including the frequency and duration of exercise interventions, and the neural circuits activated after treatment.

### 3.2 Modulation of eCBs

The eCB system is involved in a variety of processes including brain plasticity, learning and memory, injury perception, inflammation, pain, neuronal development, and the regulation of emotion and stress (Matei et al., 2023). eCB modulates pain and acts as an analgesic in acute injury receptors and in models of clinical neuropathy. The basis of the analgesic action of eCB is related to cannabinoid receptors, including cannabinoid receptors CB1 and CB2, which are coupled to Gi proteins. CB1 receptors are associated with central nervous system disorders such as multiple sclerosis and Alzheimer’s disease. CB1 receptors are primarily located in nerve endings and inhibit neurotransmitter release, affecting pain signaling in peripheral, spinal and supraspinal regions. In contrast, CB2 receptors are associated with pain and inflammation (Svízenská et al., 2008). They are primarily located in nervous system cells and tissues responsible for pain processing and in immune cells that regulate the transmission of inflammatory nociceptive hypersensitivity.

Studies in humans and rodents have shown that moderate exercise induces neuroplasticity and functional brain connectivity, increases eCB in the brain and blood, and affects cannabinoid receptors (Park and Watkins, 2022). Exercise-induced anti-inflammatory analgesia involves activating the eCB system and downstream pain control pathways (Ludtke et al., 2020). Inhibition of eCB catabolic enzymes prolongs the antinociceptive hyperalgesic effects of swimming exercises (Ludtke et al., 2020). AMPK is a downstream goal of eCB signaling, and the exercise-activated eCB system leads to increased AMPK activity (Russe et al., 2013). The AMPK-mediated antinociceptive effect may be due to the inhibition of MAPK-c-Fos signaling pathway. c-Fos is an activator and a reliable indicator of inflammation and pain response. A previous study (King-Himmelreich et al., 2017) demonstrated a significant decrease in spinal c-Fos levels in IP mice after aerobic exercise, suggesting that AMPK-c-Fos is implicated in the perception of exercise-induced analgesia. In addition, they observed that AMPKα2 knockout mice showed no anti-inflammatory pain effects after treadmill exercise, indicating the importance of this signaling pathway for exercise-induced analgesia.

Adenylate cyclase and MAPK are negatively and positively connected to the CB1 and CB2 that comprise the eCB system, respectively (Dos Santos et al., 2019). Several CB2 agonists exhibit analgesic, antinociceptive and antiabnormal pain activity in the absence of CB1-induced psychostimulant effects (Borgonetti et al., 2023). Exercise induces the spinal release of eCBs (anandamide), which activates CB2 receptors, decreases microglial expression and the secretion of pro-inflammatory substances. These substances reduce the transmission of nociceptive impulses at the site of pain, thereby producing anti-inflammatory and antinociceptive effects. Infrared thermography has shown that exercise alleviates vasodilation and paw edema in animals with IP (Dos Santos et al., 2019; Ludtke et al., 2020). Notably, eCB signaling appears to be intensity-dependent, with very high and low intensity exercise likely not significantly altering circulating eCB levels, and significant changes in circulating eCB observed only after moderate intensity (Watkins, 2018). Therefore, it is critical to understand how cannabinoid receptor signaling exerts analgesic effects to ameliorate IP under exercise conditions, which may expand our understanding of IP processes.

### 3.3 Alteration of excitability balance in the SDH

Prior to being projected to higher centers, sensory information is modulated by excitatory and inhibitory interneurons in the SDH. The excitatory balance of the SDH is altered in a CFA-induced inflammatory arthritis animal model, resulting in microglial proliferation, a substantial reduction of inhibitory ends, and reduced production of potassium chloride cotransporter 2 (Locke et al., 2020). In a rodent with inflammatory arthritis, SDH underwent peripheral and central sensitization, with increased conduction from primary afferents to SDH and elevated expression of CGRP and SP (Fernandez-Zafra et al., 2019). Exercise intervention in mice with IP reduces GFAP expression in satellite glial cells, decreases neuronal gap junction-mediated coupling in the DRG, and prevents mechanical pain (Feldman-Goriachnik et al., 2022).

Cg-induced IP models produce mechanical hyperalgesia associated with plasticity changes in SDH neurons, resulting in increased levels of immunoreactive CGRP, SP, pro-inflammatory substances, and microglia in the spinal cord. Some or all of these changes in the SDH neurons may lead to central sensitization in the spine or supraspinal column (Radhakrishnan et al., 2003). Central sensitization and enhanced sensitivity of peripheral nociceptors lead to secondary hyperalgesia. Dos Santos et al. (2019) found that swimming prevented the production of IBA1, a microglia marker, in the SDH and the release of TNF-α. Neurons in the spine that are damaged or healthy can become hyperexcitable through the action of BDNF and NGF (López-Álvarez et al., 2015). The release of neurotrophic factors stimulates the nociceptive receptors and induces nociceptive hypersensitivity. Future research should consider studying the crosstalk between exercise and neurotrophic factors that reduce IP.

Additionally, the cartilage along the vasculature is invaded by sympathetic and peptidergic nociceptive fibers, altering innervation. Following the new innervation of cartilage, nociceptive receptors are close to the changed cartilage matrix and synovial fluid, which contain pro-injurious factors inflammatory that increase NGF levels (Locke et al., 2020). However, continuous passive motion decreases CGRP expression in the superficial SDH, thereby inhibiting central sensitization caused by arthritic immobilization, restoring inflammatory primary hyperalgesia, increasing the range of motion in knee flexion, and preventing the onset of secondary pain (Nakabayashi et al., 2016). Previous studies indicated that continuous passive motion decreases IL-1β and COX-2 expression and increases IL-10 release in rat meniscal chondrocytes (Ferretti et al., 2005). These findings explain exercise affects the healing and regeneration of painful, inflamed joint tissue, which appears to depend on changes in the balance of SDH excitation and inhibition.

### 3.4 Regulation of immune cell polarization balance

Macrophages play a multifaceted function in maintaining the tissue microenvironment. They can be polarized into M1 macrophages and M2 macrophages depending on the production of enzymes, receptors, chemokines, and trophic factors (Locati et al., 2020). One mechanism by which exercise prevents and treats IP is by modulating the balance of M1 and M2 macrophages. M1 pro-inflammatory macrophages produce TNF-α and IL-1β, which induce phosphorylation of NMDARs to promote persistent pain signaling, and M2 anti-inflammatory macrophages produce IL-10. Therefore, neuroimmune mechanisms of transduction and transmission can regulate inflammatory muscle pain information from the peripheral nervous system to the CNS.

In an animal of monosodium urate-induced inflammatory gout, exercise intervention reduced inflammation and the immunomodulatory response resulting from immune tolerance. Specifically, NOD-like receptor family 3 inflammasome activation in an inflammatory gout mouse leads to the overproduction of mitochondrial reactive oxygen species (So and Martinon, 2017), contributing to IL-1β secretion through TLR2 signaling in concert with the activation of ASC-caspase-1 (Furrer et al., 2017). In mice with gout, there is a significant decrease in F4/80^+^ macrophage and MPO^+^ neutrophil infiltration in the synovium after low-intensity and moderate-intensity aerobic exercise (Furrer et al., 2017). Exercise decreased the production of TLR2 and the pro-inflammatory chemokine CXCL1 on the surface of cells. Therefore, exercise may block the activation of NOD-like receptor family 3 inflammasomes, an immune response that is a long-term, small-scale inflammatory response induced by immune tolerance.

PPARγ exerts antioxidant and anti-inflammatory effects by blocking the MAPK/NF-κB. Activation of PPARγ promotes macrophage polarization toward M2 and increases the secretion of myokines, cytokines, and chemokines (Kim et al., 2017; de Azambuja et al., 2020). Pharmacological and immunochemical experiments confirm that exercise inhibits the development of persistent inflammatory pain in rodents through activating PPARγ and its downstream pathways (de Azambuja et al., 2021). Specifically, exercise boosts the proportion of F4/80^+^-CD206^+^ macrophages (anti-inflammatory) and decreases the proportion of F4/80^+^-CD11c^+^ macrophages (pro-inflammatory), supporting the possibility of macrophages and skeletal muscle cells interacting (Pillon et al., 2013). Prior research has also demonstrated that PPARγ is an effective target for reducing IP by downregulating the expression of CINC-1 (pro-inflammatory). These findings suggest that physical exercise can rapidly adjust the immune response to produce an anti-inflammatory milieu by balancing M1 and M2 macrophages. In addition, Lee et al. (2022) found that the expression of CD206, an M2 macrophage marker, was significantly upregulated, IL-1Ra expression was increased, and overall the secretion of pro-inflammatory factors was downregulated in the intervertebral discs of voluntarily running SPARC-null female rodents compared to those in sedentary wild-type rodents and sedentary SPARC-null rodents. Interestingly, the levels of cytokines and chemokines were similar between SPARC-null male rodents that voluntarily ran and sedentary SPARC-null rodents. Specifically, the mRNA levels of *ITGAM*, an indicator of overall macrophages, were upregulated in exercising animals, but M1 and M2 mRNA indicators showed no change in levels. SPARC-null male rodents have higher concentrations of M-CSF and VEGF, which could explain the enhanced ITGAM macrophage differentiation (Lee et al., 2022). The sexually dimorphic effect of exercise on inflammatory disc pain may be due to different macrophage responses.

### 3.5 Alterations in cytokines

Cytokines are intercellular signaling molecules that regulate inflammation, neuropathy, cancer, and pain. The balance between pro-inflammatory and anti-inflammatory cytokines is a key concept in understanding the effects of the immune system on chronic pain. Exercise increases muscle and blood levels of IL-10, an anti-nociceptive cytokine that acts by inhibiting the production of nociceptive cytokines such as IL-1β, IL-6, IL-8, and TNF-α, which can reduce the hypersensitivity of injury receptors. Leung et al. (2016) showed that blockade of IL-10 receptors throughout the body or locally in muscles prevented the analgesic effects of regular physical activity. Similarly, administration of IL-10 to sedentary mice prevented the development of pain sensitization in muscle, mimicking the protective effects of regular physical activity.

IL-16, a cytokine with chemotactic properties, is produced by immune cells (microglia, monocytes/macrophages, T cells, and dendritic cells) and neurons. Zhu et al. (2024) induced an IP model by injecting 10 μL of 50% CFA into the left footpad under isoflurane anesthesia, and identified IL-16 as a differentially expressed gene in the dorsal horn of the spinal cord of IP mice by RNA sequencing. Their further analysis revealed that IL-16-CD4 signaling triggers pain and activates microglia and astrocytes in the dorsal horn of the spinal cord, leading to IP. Given the wide-ranging effects of IL-16 on microglia and T cells, there is a necessity to explore the possibility that exercise by decreasing IL-16 in the SDH may serve as a therapeutic target for IP (Wu et al., 2021).

### 3.6 Role of glial cells

Neuroinflammation is a complex and well-coordinated process consisting of various glial cells and peripheral immune cells in the central nervous system (CNS). Astrocytes and microglia are two important glial cells that are triggered by peripheral tissue and nerve injury to release pro-inflammatory cytokines (IL-1β, IL-6, and TNF-α), which may be involved in chronic pain (Al-Zaidi et al., 2021). In addition, activated microglia (IBA1) generate important mediators of chronic and persistent pain by modulating synaptic function and connectivity in the spinal cord (Takahara-Yamauchi et al., 2021).

Despite the absence of running-wheel exercise in environmental enrichment (EE), the procedure enables more physical activity and can stimulate natural behaviors in animals (Kempermann, 2019). An EE study analyzed injurious and inflammatory responses in a mouse model of arthritis. EE reduced immunopositivity for the microglia marker IBA1 in the prefrontal cortex of mice, as well as reduced inflammatory scores and acute edema formation (Estrázulas et al., 2022). Furthermore, EE reduced TNF-α and IL-1β and increased BDNF expression in mice (Zhang and Lee, 2018). Numerous studies have focused on BDNF/TrkB signaling-mediated generation of persistent pain. BDNF is a neurotrophic factor involved in dendritic spine formation, survival of newborn neurons in the hippocampus, and has a significant effect on learning and memory. BDNF is regulated by neuronal activity and stored in dense core synaptic vesicles at the terminals of neurons, and is overexpressed and released from neurons when subjected to nerve injury or inflammation. This process leads to synaptic plasticity and central sensitization, which are involved in the development of chronic pain.

Interstitial cystitis/bladder pain syndrome (IC/BPS) is a chronic inflammatory disease of the bladder accompanied by bladder pain. Studies have shown that BDNF may promote the activation of astrocytes and microglia through the BDNF-TrkB- p38/JNK signaling pathway, releasing large amounts of pro-inflammatory factors, which consequently exacerbate the neuroinflammatory and mechanically abnormal pain of cystitis (Ding et al., 2020). Several studies have found that voluntary exercise improves nociceptive hypersensitivity and voiding function in IC/BPS and increases BDNF levels (Pierce et al., 2018; Sanford et al., 2020).

Takahara-Yamauchi et al. (2021) analyzed whether voluntary exercise produces raw analgesic effects in relation to glial cells. Specifically, voluntary exercise produced analgesic effects in a rat model of IP by inhibiting the proliferation of spinal microglia, up-regulating TrkB and down-regulating KCC2, compared to the sedentary group. However, this study showed no change in BDNF levels after exercise, possibly because formalin injection enhanced BDNF release from microglia without affecting the apparent total amount of BDNF.

## 4 Conclusion

This review explores the potential mechanisms of exercise on IP, offering a qualitative analysis of the efficacy of exercise on IP with good anti-inflammatory and analgesic effects. This can be achieved through modulation of synaptic plasticity in the ACC, endogenous cannabinoids, excitatory balance in the SDH, immune cell polarization balance, cytokines, and glial cells. Studying the cellular and molecular mechanisms by which exercise prevents and alleviates IP will help develop exercise prescriptions and utilization rates for treating patients with IP and improve the implementation and compliance of exercise as an essential intervention for IP. Although 150 min of moderate physical activity per week or 2 days of strengthening exercise per week is beneficial, it is not clear whether this dose is needed for pain relief. From a clinical perspective, randomized controlled trials are needed to further translate the preclinical evidence base into the clinical arena. We would like to conduct a quantitative analysis of the effects of exercise on IP, such as examining the specific type of exercise (swimming, aerobic, and endurance training) and effective dose (frequency, duration, and the number of sessions).

Although our literature review suggests that all exercise modalities have beneficial effects, most animal model studies are based on voluntary exercise and swimming, rather than strength training typically performed in a rehabilitative setting. In addition, whether resistance training-based exercise programs have the same mechanisms for producing analgesia deserves further investigation. Sex differences exist in nociceptive hypersensitivity mechanisms and pain phenotypes, and most studies have been conducted using male animals. Future work needs to include both male and female animals to determine if exercise-induced analgesic mechanisms work equally well. The relationship between biomolecules involved in IP mechanisms is complex, and many biomolecules are involved in both central and peripheral sensitization, forming a network of pain mechanisms through synergistic and inhibitory relationships. It is necessary to explore the mechanism of IP from multiple molecular targets, pathways, and levels in future studies.

## Author contributions

MW: Writing – original draft. WS: Data curation, Methodology, Writing – review & editing. MZ: Methodology, Visualization, Writing – review & editing. LT: Investigation, Methodology, Writing – review & editing. QT: Conceptualization, Validation, Supervision, Writing – review & editing. LZ: Conceptualization, Funding acquisition, Supervision, Writing – review & editing.
